# Development and validation of a risk prediction model for chronic kidney disease among individuals with type 2 diabetes

**DOI:** 10.1038/s41598-022-08284-z

**Published:** 2022-03-21

**Authors:** Cheng-Chieh Lin, May Jingchee Niu, Chia-Ing Li, Chiu-Shong Liu, Chih-Hsueh Lin, Shing-Yu Yang, Tsai-Chung Li

**Affiliations:** 1 School of Medicine, College of Medicine, China Medical University, Taichung, Taiwan; 2 Department of Family Medicine, China Medical University Hospital, Taichung, Taiwan; 3 Department of Medical Research, China Medical University Hospital, Taichung, Taiwan; 4 Department of Public Health, College of Public Health, No. 100, Sec. 1, Jingmao Rd., Beitun Dist., Taichung City, 406040 Taiwan; 5 Department of Healthcare Administration, College of Medical and Health Science, Asia University, Taichung, Taiwan

**Keywords:** Endocrinology, Nephrology, Risk factors

## Abstract

Many studies had established the chronic kidney disease (CKD) prediction models, but most of them were conducted on the general population and not on patients with type 2 diabetes, especially in Asian populations. This study aimed to develop a risk prediction model for CKD in patients with type 2 diabetes from the Diabetes Care Management Program (DCMP) in Taiwan. This research was a retrospective cohort study. We used the DCMP database to set up a cohort of 4,601 patients with type 2 diabetes without CKD aged 40–92 years enrolled in the DCMP program of a Taichung medical center in 2002–2016. All patients were followed up until incidences of CKD, death, and loss to follow-up or 2016. The dataset for participants of national DCMP in 2002–2004 was used as external validation. The incident CKD cases were defined as having one of the following three conditions: ACR data greater than or equal to 300 (mg/g); both eGFR data less than 60 (ml/min/1.73 m^2^) and ACR data greater than or equal to 30 (mg/g); and eGFR data less than 45 (ml/min/1.73 m^2^). The study subjects were randomly allocated to derivation and validation sets at a 2:1 ratio. Cox proportional hazards regression model was used to identify the risk factors of CKD in the derivation set. Time-varying area under receiver operating characteristics curve (AUC) was used to evaluate the performance of the risk model. After an average of 3.8 years of follow-up period, 3,067 study subjects were included in the derivation set, and 786 (25.63%) were newly diagnosed CKD cases. A total of 1,534 participants were designated to the validation set, and 378 (24.64%) were newly diagnosed CKD cases. The final CKD risk factors consisted of age, duration of diabetes, insulin use, estimated glomerular filtration rate, albumin-to-creatinine ratio, high-density lipoprotein cholesterol, triglyceride, diabetes retinopathy, variation in HbA1c, variation in FPG, and hypertension drug use. The AUC values of 1-, 3-, and 5-year CKD risks were 0.74, 0.76, and 0.77 in the validation set, respectively, and were 0.76, 0.77, and 0.76 in the sample for external validation, respectively. The value of Harrell’s c-statistics was 0.76 (0.74, 0.78). The proposed model is the first CKD risk prediction model for type 2 diabetes patients in Taiwan. The 1-, 3-, and 5-year CKD risk prediction models showed good prediction accuracy. The model can be used as a guide for clinicians to develop medical plans for future CKD preventive intervention in Chinese patients with type 2 diabetes.

## Introduction

Diabetes is a significant worldwide health threat, and the World Health Organization reported diabetes as the seventh leading cause of death in 2016^1^. The global age-standardized prevalence of diabetes has almost doubled since 1980, rising from 4.7% to 8.5% in the adult population; in 2014, an estimated 422 million adults lived with diabetes compared with the 108 million in 1980. The global costs of diabetes health care are a huge economic burden. Diabetes causes numerous comorbidities, including chronic kidney disease (CKD). The prevalence of recognized CKD has steadily risen worldwide, driven by demographic factors, such as age and population growth^2^. Taiwan has the highest incidence and prevalence of end-stage renal disease in the world^3^ and chronic kidney disease (CKD) is the key factor contributing to this burden. It has been reported that prevalence of CKD stages 3–5 ranged from 6.9%^4^ in 1999–2009 to 9.06%^5^ in 2002 in various community-based populations; and the corresponding prevalence for total CKD stages were 11.9%^4^ and 15.46%^6^, respectively. The CKD prevalence in Taiwan was high, varying according to age, gender, and comorbidities of hypertension, diabetes, and metabolic^6^. However, it has been reported that the awareness of CKD was low with prevalence of 8%, 25%, and 71.4% for persons with stage 3, 4, and 5, respectively^5^.

The development of a prediction model for incident CKD in patients with type 2 diabetes may facilitate the prevention of CKD incidence in this patient population. This goal may avoid the expenditure that is associated with CKD and may consequently reduce ESRD cases. In addition, predicting CKD in patients with type 2 diabetes can aid in specific screening of high-risk patients, guiding and planning of preventative interventions, and arrangement future health care needs. CKD risk models had been set up for the general population^7–10^ and various ethnic groups, such as white^8^, black^9^, and Asian or others^11^. Although persons with diabetes share most of the same risk factors, such as obesity, blood pressure, and renal markers, with those without diabetes^12–15^, the prediction models for general populations cannot consider diabetes-related measures. However, no prior study built a prediction model for type 2 diabetes patients exclusive for Chinese ethnicity.

The most important biomarkers for diabetes care are glycemic control and variation in glycemic markers^16–19^. CKD risk prediction models for patients with type 2 diabetes built in prior studies considered traditional factors, such as age, sex, blood pressure, renal-related factors of estimated glomerular filtration rate (eGFR), albumin-to-creatinine ratio (ACR), and diabetes related-factors^9,11,20–25^. Most studies considered biomarkers, including HbA1c level, but not the variation in glycated hemoglobin (HbA1c) or fasting plasma glucose (FPG) , even though prior studies reported that visit-to-visit variation in HbA1c^17,18^ and FPG^18^ was associated with CKD incidence. In this study, we aimed to establish a prediction model for CKD by considering glycemic variation and traditional risk factors in patients with type 2 diabetes by using the Diabetes Care Management Program (DCMP) database of a medical center in Taiwan. This prediction model only considers risk factors that have been generally monitored in clinical practice and are precisely measured to ensure its feasibility and accuracy for clinical application.

## Results

These 4,601 participants were randomly allocated to a derivation (n = 3,067) or validation set (n = 1,534) at a 2:1 ratio (Supplementary Fig. 1). Table 1 shows the baseline characteristics of the study subjects in the derivation set of 3,067 patients with type 2 diabetes and in the validation set of 1,534 patients. After an average of 3.8 years of follow-up period, 786 (25.63%) and 378 (24.64%) newly diagnosed CKD cases were included in derivation and validation sets, respectively. The mean values (standard deviation) for the number of BP, HbA1c, and FPG in the derivation set were 4.96 (2.56), 6.17 (2.02), and 9.32 (5.86), respectively; and the corresponding values in the validation set were 5.02 (2.53), 6.22 (1.99), and 9.79 (6.35), respectively. The standardized effect size values of all baseline variables and outcome variables were less than 0.1, demonstrating that the variables in both sets had similar distributions.

**Table 1:** Baseline characteristic of study population according to derivation and validation sets.

Variables	Derivation set (n = 3,067) Mean ± SD or n (%)	Validation set (n = 1,534) Mean ± SD or n (%)	Standardized effect size
**Socio-demographic factors and behaviors**			
Age (year)	59 ± 10	59 ± 10	− 0.02
*Gender*			
Female	1430 (47)	747 (49)	− 0.04
Male	1637 (53)	787 (51)	0.04
Smoking habit	515 (17)	235 (15)	0.04
Alcohol habit	258 (8)	125 (8)	0.01
Exercise habit	1899 (62)	965 (63)	− 0.02
BMI (kg/m^2^)	26 ± 4	26 ± 4	0.01
Nutritional intake			
*Fat*			
Sufficient	2608 (88)	1315 (89)	− 0.02
Over intake	281 (10)	142 (10)	0.00
Insufficient	65 (2)	23 (1)	0.05
*Protein*			
Sufficient	2665 (90)	1353 (91)	− 0.04
Over intake	162 (6)	82 (6)	0.00
Insufficient	127 (4)	45 (3)	0.07
*Carbohydrate*			
Sufficient	2665 (90)	1344 (91)	− 0.02
Over intake	145 (5)	69 (5)	0.01
Insufficient	144 (5)	67 (4)	0.02
Age of diabetes (years)	54 ± 11	55 ± 10	− 0.01
Duration of diabetes (years)	5 ± 6	5 ± 6	− 0.02
**Blood pressures**			
SBP (mm Hg)	132 ± 16	132 ± 16	− 0.01
DBP (mm Hg)	81 ± 11	81 ± 11	0.01
**Glycemic factors**			
HbAlc level (%)	8 ± 2	8 ± 2	0.01
FPG (mm Hg)	147 ± 49	146 ± 48	0.02
**Lipid profiles**			
LDL-C (mg/dL)	109 ± 34	109 ± 33	0.02
HDL-C (mg/dL)	44 ± 12	44 ± 12	0.00
Triglyceride (mg/dL)	156 ± 155	154 ± 169	0.02
Total cholesterol (mg/dL)	184 ± 40	183 ± 40	0.03
**Renal function markers**			
Creatinine (mg/dL)	1 ± 0	1 ± 0	0.01
eGFR (ml/min/1.73m^2^)	92 ± 16	92 ± 16	0.02
ACR (mg/g)	27 ± 44	27 ± 43	0.00
**Variation in BP and glycemic factors**			
Variation of SBP (%)	7 ± 4	7 ± 3	− 0.01
Variation of DBP (%)	7 ± 4	8 ± 4	− 0.03
Number of BP measurements	5 ± 3	5 ± 3	− 0.02
Variation of Hba1c (%)	8 ± 7	8 ± 7	0.00
Number of HbA1c measurements	6 ± 2	6 ± 2	− 0.03
Variation of FPG (%)	18 ± 12	18 ± 13	− 0.01
Number of FPG measurements	9 ± 6	10 ± 6	− 0.08
**Baseline cardiovascular diseases**			
Hypertension	749 (24)	419 (27)	− 0.07
*BP abnormality*			
≤ 140/90 mmHg	2091 (68)	1040 (68)	0.01
> 140/90 mmHg	976 (32)	494 (32)	− 0.01
Hyperlipidemia	412 (13)	216 (14)	− 0.02
Stroke	94 (3)	39 (3)	0.03
Ischemic heart disease	127 (4)	61 (4)	0.01
Angina	53 (2)	25 (2)	0.01
**Baseline diabetes-related disorders or complications**			
Obesity	541 (18)	271 (18)	0.00
Peripheral neuropathy	148 (5)	70 (5)	0.01
Diabetes neuropathy	38 (1)	36 (2)	− 0.09
Diabetes retinopathy	600 (20)	325 (21)	− 0.04
Diabetic ketoacidosis	5 (1)	4 (1)	− 0.02
Hypoglycemia	9 (1)	2 (1)	0.03
Postural hypotension	60 (2)	31 (2)	0.00
HHNK	11 (1)	7 (1)	− 0.02
Vascular bypass	1 (1)	0 (0)	0.02
Peripheral vascular disease	3 (1)	1 (1)	0.01
Amputation	10 (1)	3 (1)	0.02
**Medication use**			
*Oral medication agents*			
No	231 (8)	102 (7)	0.03
Yes	2836 (92)	1432 (93)	− 0.03
Sulfonylurea	807 (26)	418 (27)	− 0.02
Meglitinide	69 (2)	49 (3)	− 0.06
Biguanide	1332 (43)	661 (43)	0.01
α-Glucosidase inhibitor	163 (5)	91 (6)	− 0.03
Insulin sensitizer	62 (2)	27 (2)	0.02
DPP4 inhibitor	114 (4)	63 (4)	− 0.02
Compound	338 (11)	168 (11)	0.00
*Insulin injection*			
No	2822 (92)	1432 (93)	− 0.05
Yes	245 (8)	102 (7)	0.05
Aspart	10 (1)	1 (1)	0.05
RI	76 (2)	24 (2)	0.06
NPH	88 (3)	34 (2)	0.04
Levemir	27 (1)	10 (1)	0.03
Lantus	32 (1)	16 (1)	0.00
Glp-1 analogue	1 (1)	0 (0)	0.02
Anti-hypertension medications	1125 (37)	607 (40)	− 0.06
Cardiovascular medications	522 (17)	261 (17)	0.00
Hyperlipidemia medications	450 (15)	245 (16)	− 0.04
**Outcome**			
CKD			
No	2281 (74)	1156 (75)	− 0.02
Yes	786 (26)	378 (25)	0.02

The standardized effect size defined as the difference of two population means (or proportions) and it is divided by the standard deviation.

* ACR* albumin to creatinine ratio,* variation of SBP* variation of systolic blood pressure,* variation of DBP* variation of diastolic blood pressure,* variation of FPG* variation of fasting plasma glucose,* SBP* systolic blood pressure,* DBP* diastolic blood pressure,* FPG* fasting plasma glucose,* LDL-C* Low-density lipoprotein,* HDL-C* High-density lipoprotein,* GPT* Glutamic Pyruvic Transaminase,* eGFR* estimate glomerular filtration rate.* CKD* chronic kidney disease,* HHNK* Hyperglycemic hyperosmolar nonketotic coma.

Supplementary Table S1 shows the number of participants, CKD cases, person-years of follow up, incidence rates of CKD, age- and gender-adjusted HR, and the p-value for baseline predictors in the derivation set. All variables with *p* < 0.25 were entered in the multivariate model simultaneously and backward deleted if they were not statistically significant.

Table 2 presents the final multivariate Cox proportional hazards model for CKD in persons with type 2 diabetes, comprising significant risk factors, such as age, duration of diabetes, insulin use, eGFR, ACR, high-density lipoprotein cholesterol (HDL-C), triglyceride (TG), diabetes retinopathy, variation of HbA1c, variation of FPG, and hypertension drug use. The interaction terms between any two of these risk factors were assessed one by one. We detected no significant interaction.

**Table 2:** Parameter estimates of regression coefficient and the means or proportions of predictors for CKD from the final multivariate Cox’s proportional hazards model.

Risk factor	$\widehat{{\varvec{\beta}}}$($\widehat{{\varvec{S}}{\varvec{E}}}$)	Mean or proportion	HR (95% CI)	p-value
**Socio-demographic factors**				
Age	0.03 (0.01)	59.23	1.03 (1.03, 1.04)	< 0.001
Duration of type 2 diabetes (years) (< 1 year as reference)				
1–5	0.06 (0.10)	0.45	1.06 (0.86, 1.30)	0.56
> 5	0.24 (0.11)	0.30	1.28 (1.03, 1.58)	0.02
**Renal function markers**				
eGFR (≥ 90 mL/min/1.73m^2^ as reference)	0.84 (0.08)	0.40	2.31 (1.96, 2.71)	< 0.001
ACR (< 30 mg/g as reference)	1.05 (0.07)	0.23	2.87 (2.48, 3.31)	< 0.001
**Lipid profiles**				
HDL-C (male ≥ 40 / female ≥ 50 mg/dl as reference)	0.18 (0.08)	0.56	1.20 (1.03, 1.39)	0.02
Triglyceride (< 150 mg/dL as reference)	0.31 (0.08)	0.37	1.37 (1.18, 1.59)	< 0.001
**Variation in glycemic factors**				
Variation of HbA1c (%), (≤ 3.9% as reference)				
4.0–8.0	0.19 (0.09)	0.33	1.22 (1.01, 1.46)	0.04
> 8.0	0.31 (0.10)	0.33	1.36 (1.11, 1.67)	0.003
Variation of fasting plasma glucose (%), (≤ 10.7% as reference)				
10.8–20.3	0.10 (0.10)	0.33	1.10 (0.90, 1.34)	0.34
> 20.3	0.34 (0.11)	0.33	1.40 (1.14, 1.73)	0.002
**Baseline diabetes-related disorders or complications**				
Diabetes retinopathy	0.29 (0.09)	0.20	1.34 (1.13, 1.58)	< 0.001
**Medication use**				
Insulin use	0.39 (0.11)	0.08	1.50 (1.21, 1.86)	< 0.001
Hypertension drug use	0.20 (0.07)	0.37	1.20 (1.04, 1.38)	0.005

*CKD* chronic kidney disease,* eGFR* estimate glomerular filtration rate,* ACR* albumin to creatinine ratio,* HDL-C* High-density lipoprotein,* variation of FPG* variation of fasting plasma glucose,* HR* hazard ratio,* CI* confidence intervals.

We assigned the points for each factor in the final multivariate model (Table 3). Supplementary Table S2 shows the predicted risks for all possible sum of scores across all predictors**.** In the study, the possible sum of score ranged from 0 to 37. We estimated the 1-, 3-, and 5-year risks of CKD for each sum point. The risks of CKD were from 1.08% to 99.52% for 1-year risk, 2.96% to 100% for 3 years, and 4.54% to 100% for 5 years. Sensitivity, specificity, positive predictive value (PPV) and negative predictive value (NPV) were calculated for cutoff points at 5%, 10%, 20%, 30%, 40% and 50% of risks to define high risks for CKD (Table 4).

**Table 3:** To calculate the number of points for each category of main effects.

Risk factor	Categories	Reference value (W_ij_)	${\beta }_{i}$	${\beta }_{i}({W}_{ij}-{W}_{iREF})$	Points_ij_ = ${\beta }_{i}({W}_{ij}-{W}_{iREF})/B$
**Socio-demographic factors**					
Age			0.03		
	40–44	42 = W_1REF_		0	0
	45–49	47		0.15	1
	50–54	52		0.30	2
	55–59	57		0.45	3
	60–64	62		0.60	4
	65–69	67		0.75	5
	70–74	72		0.90	6
	75–79	77		1.05	7
	80–84	82		1.20	8
	85–89	87		1.35	9
	90–94	92		1.50	10
Duration of type 2 diabetes					
	0	0 = W_2REF_		0	0
	1–5	1	0.06	0.06	0
	> 5	2	0.24	0.24	2
**Renal function markers**					
eGFR (mL/min/1.73 m^2^)					
	≥ 90	0 = W_3REF_		0	0
	< 90	1	0.84	0.84	6
ACR (mg/g)					
	< 30	0 = W_4REF_			0
	≥ 30	1	1.05	1.05	**7**
**Lipid profiles**					
HDL-C (mg/dl)					
	≥ 40 (male) ≥ 50 (female)	0 = W_5REF_		0	0
	< 40 (male) < 50 (female)	1	0.18	0.18	1
Triglyceride (mg/dl)					
	< 150	0 = W_6REF_		0	0
	≥ 150	1	0.31	0.31	2
**Variation in glycemic factors**					
Variation of HbA1c (%)					
	≤ 3.9	0 = W_7REF_		0	0
	4.0–8.0	1	0.19	0.19	1
	> 8.0	2	0.31	0.31	2
Variation of fasting plasma glucose (%)					
	≤ 10.7	0 = W_8REF_		0	0
	10.8–20.3	1	0.10	0.10	1
	> 20.3	2	0.34	0.34	2
**Baseline diabetes-related disorders or complications**					
Diabetes retinopathy					
	No	0 = W_9REF_		0	0
	Yes	1	0.29	0.29	2
**Medication use**					
Insulin use					
	No	0 = W_10REF_		0	0
	Yes	1	0.39	0.39	2
Anti-hypertension drug use					
	No	0 = W_11REF_		0	0
	Yes	1	0.20	0.20	1

**Table 4:** Risk classification of sensitivity, specificity, PPV, and NPV.

Cutoff point for high risk	Risk scores	Sensitivity	Specificity	PPV	NPV	N (%)			
						Patients classified as high risk	Patients classified as high risk who develop CKD	Patients classified as low risk	Patients classified as low risk who develop CKD
5%	1	100	0	25	100	4590 (100)	1164 (25)	11 (1)	0 (0)
10%	5	98	12	27	94	4169 (91)	1139 (27)	432 (99)	25 (6)
20%	10	87	47	36	91	2835 (62)	1012 (36)	1766 (38)	152 (9)
30%	13	76	64	41	89	2141 (47)	888 (41)	2460 (53)	276 (11)
40%	15	65	75	47	86	1626 (35)	759 (47)	2975 (65)	405 (14)
50%	17	52	85	54	84	1123 (24)	603 (54)	3478 (76)	561 (16)

*PPV* Positive predictive value,* NPV* Negative predictive value,* CKD* chronic kidney disease.

**Figure 1:**
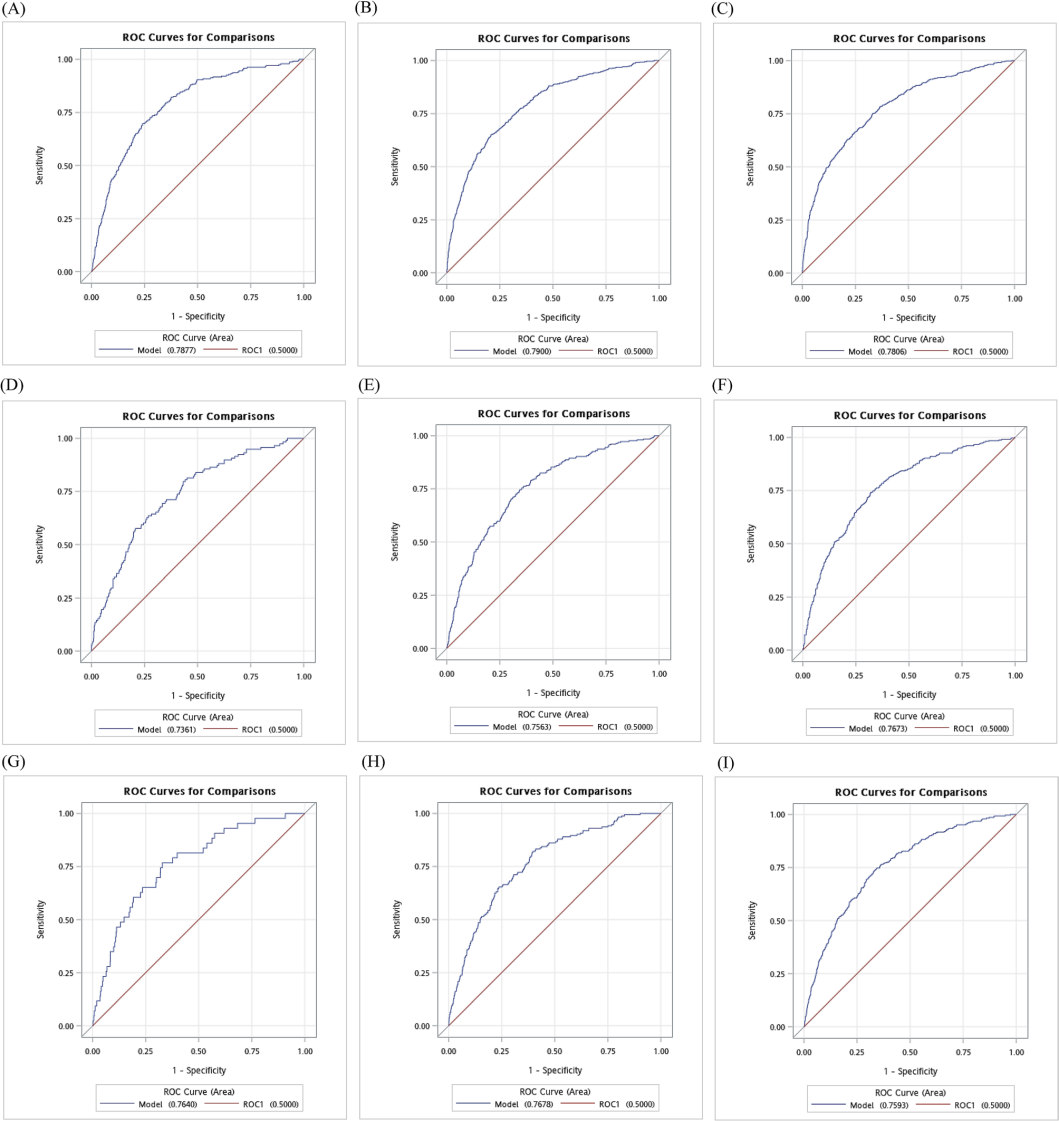
Receiver operating characteristic curves (ROCs) for 1-year (**A**), 3-year (**B**), and 5-year (**C**) CKD risk in derivation set, 1-year (**D**), 3-year (**E**), and 5-year (**F**) CKD risk in validation set, and 1-year (G), 3-year (**H**), and 5-year (**I**) CKD risk in the sample for external validation.

Figure 1 shows the AUC for 1-, 3-, and 5-year CKD risks in the derivation and validation sets as well as in the sample for external validation. The AUCs for 1-, 3-, and 5-year CKD risks in the derivation set were 0.79, 0.79, and 0.78, respectively. The values reached 0.74 (1 year), 0.76 (3 years), and 0.77 (5 years) in the validation set and the corresponding values were 0.76, 0.77, and 0.76 in the sample for external validation. All the AUC values in the derivation and validation sets indicate that our prediction model had good discrimination capability. Harrell’s c-statistics for time-varying ROC was 0.76 (0.74–0.78).

**Figure 2:**
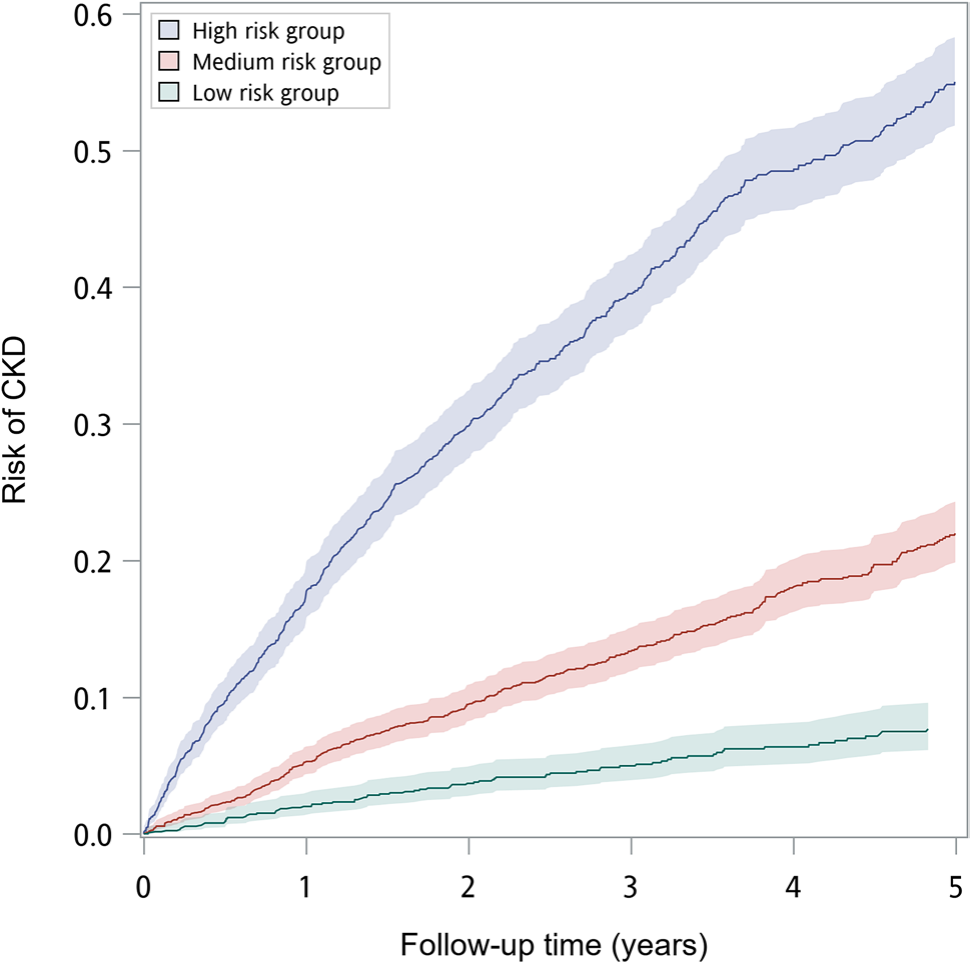
The 5-year cumulative incidence of low, median, and high groups from validation set.

We used tertiles as cutoff points to categorize the sum risk scores and grouped the subjects with type 2 diabetes into low-, median-, and high-risk groups in the validation set to compare their 5-year incident density rates. The incidence density rates of low-, median-, and high-risk groups were 13.9, 42.2, and 151.8 per 1,000 person-years, respectively, and showed significant difference (*p* < 0.001). The results of Kaplan–Meier analysis depict that patients in the highest tertile group had the highest risk of CKD compared with the other groups (Fig. 2).

**Figure 3:**
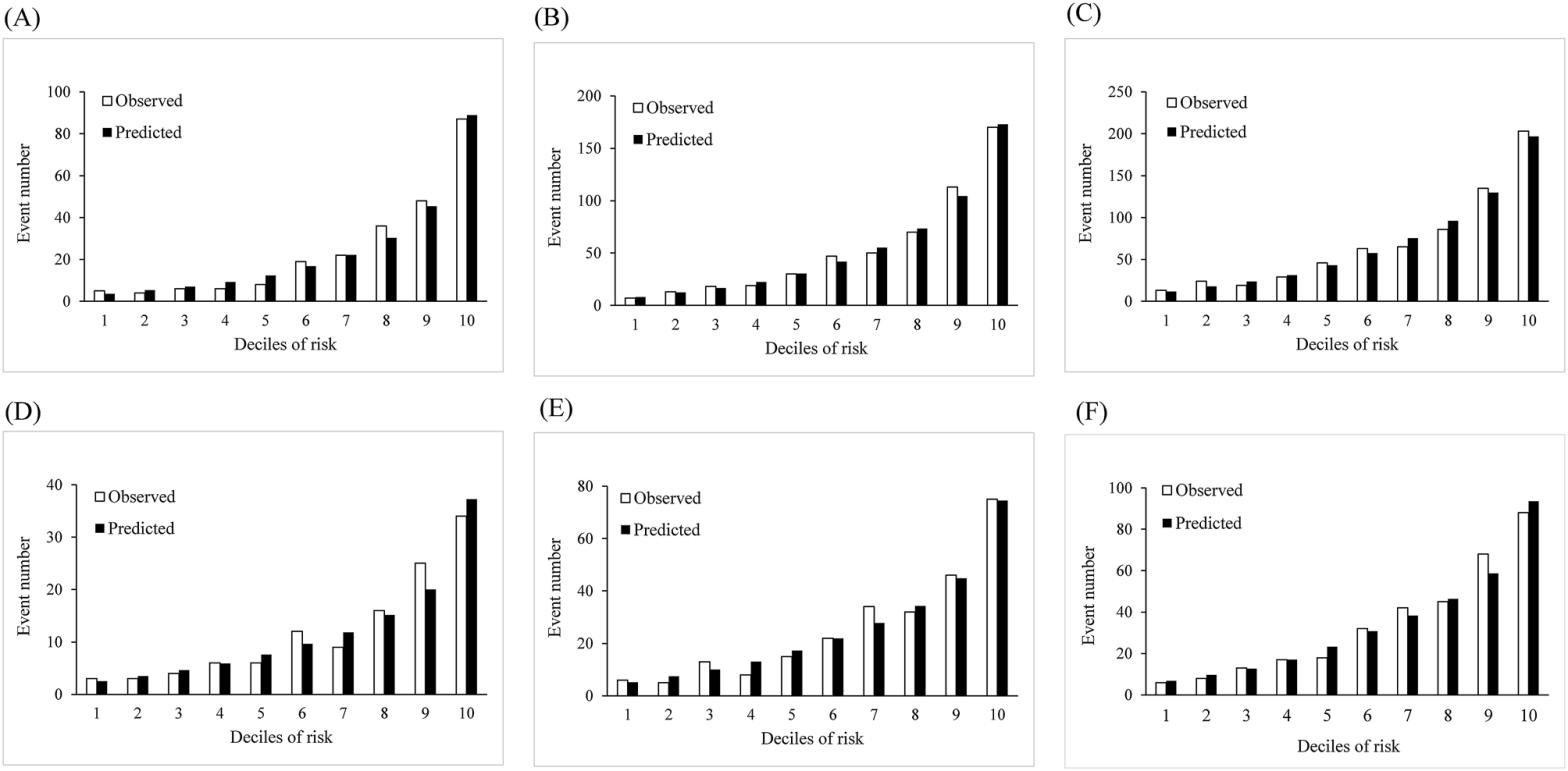
Predicted versus observed CKD cases according to deciles of 1-year (**A**), 3-year (**B**), and 5-year (**C**) CKD risk in derivation set and 1-year (**D**), 3-year (**E**), and 5-year (**F**) CKD risk in validation set.

Figure 3 shows the calibration plots comparing the actual deciles with the predicted 1-, 3-, and 5-year risks of CKD events in the derivation and validation sets. The predicted numbers of CKD based on the deciles were similar to the observed CKD numbers in 1-, 3-, and 5-year prediction in the derivation and validation sets. The Hosmer–Lemeshow $\mathcal{X}^2$ tests of the derivation set all indicated the excellent goodness of fit in the derivation and validation sets (all p values > 0.05).

The internal validation approach we used to evaluate the model performance was based on the 1000 samples from the bootstrap resampling approach. The optimism-corrected calibration intercept was − 0.0270 with a mean absolute error of 0.0086, and the corresponding slope was 0.6941 with a mean absolute error of 0.0252. The intercept and slope statistics indicated good calibration for the present model, whereas our model slightly underestimated the CKD risks.

We compared CKDPC’s equation with our scoring system using all the participants, and we used net reclassification improvement (NRI) to quantify how well our new model reclassifies subjects—either appropriately or inappropriately—compared with the CKDPC model. Supplementary Table S3 summarizes the reclassification for subjects with and without CKD events. In the 1,063 individual who experienced CKD events, our score model’s reclassification improved. The results demonstrate that our score model system provided an improvement over CKDPC’s equation with a NRI of 0.189 (*p* < 0.001). The IDI values summarize the extent of the new model in increased risk of CKD events and decreased risk of CKD non-events, and the IDI value was 0.174 (*p* < 0.001), indicating that our system improved its discriminatory capability relative to CKDPC’s equation.

Sensitivity analysis was conducted under the condition that we used multiple-imputation method to impute the missing data of the duration of diabetes, insulin injection, eGFR, ACR, HDL-C, retinopathy, TG, FPG variation, HbA1c variation, and hypertension drug use, resulting in the inclusion of 7,552 subjects in our risk model to validate the model performance. Supplementary Fig. 2 shows the results of AUC values of 0.74, 0.75, and 0.75 for 1-, 3-, and 5-year CKD in the sensitivity analysis, respectively. These results were similar to the original findings, indicating the robustness of our prediction model. The Hosmer–Lemeshow $\mathcal{X}^2$ tests in the sensitivity analysis demonstrated an excellent goodness of fit (all *p* values > 0.05) (Supplementary Fig. 3). In general, the results of sensitivity analysis demonstrate that our study’s findings were insensitive to the problem of missing data.

In the sensitivity analysis regarding the effect modification of sex on the relationships of the other risk factors with CKD incidence, no significant effect modification is found. The results for sex stratification show that the parameter estimates of factors are similar (Supplementary Table S4) although diabetes duration and HDL are borderline significant in both men and women, and variation in HbA1c is borderline significant in women. It may be due to smaller sample size after stratification.

## Discussion

### Principle findings

This research is a retrospective cohort study consisting of 4,601 persons with type 2 diabetes aged 40–92 years old. To our knowledge, this work is the first study to develop a model that predicts the CKD risks for persons with type 2 diabetes in Taiwan. The risk factors comprised age, duration of diabetes, eGFR, ACR, HDL-C, TG, variation in HbA1c and FPG, insulin use, diabetes retinopathy, and hypertension drug use. The sum risk scores ranged from 0 to 37, with 1-year risks of CKD ranging from 1.08% to 99.52%, 3-year risks from 2.96% to 100%, and 5-year risks from 4.54% to 100%. Our CKD prediction model had good discrimination capability. The AUC of 1-, 3-, and 5-year risks of CKD in the derivation set were 0.79, 0.80, and 0.78, respectively. The values were 0.74 (1 year), 0.76 (3 years), and 0.77 (5 years) in the validation set. Using national sample of persons with type 2 diabetes as external validation, the corresponding AUC values of external validation were similar to those in the derivation and validation sets, which were 0.76, 0.77, and 0.76, respectively.

### Comparisons with other studies

Three CKD models had been developed in persons with type 2 diabetes^9,11,20^. A Canadian model used the database of two randomized control trials including persons with type 2 diabetes aged ≥ 55 years, and the models of laboratory and clinical models^11^ were separately developed for CKD event and defined by new micro- or macroalbuminuria, doubling of creatinine, or ESRD. The laboratory model consisted of five predictors, such as sex in females, age, eGFR, ACR, and albuminuria stage, with an AUC of 0.68. In addition to the five predictors in the laboratory model, the clinical model comprised additional nine predictors, including race, diabetes duration, LDL-C fasting, waist circumference, major atherosclerotic cardiac events, laser therapy for diabetic retinopathy, peripheral artery disease, stroke or transient ischemic attack, and antihypertensive drugs, resulting in an AUC of 0.69. A multinational model of dataset from CKD Prognosis Consortium comprised 781,627 persons with diabetes for CKD incidence determined by an eGFR less than 45 mL/min/1.73 m^2^ and nine predictors, including old age, female, black race, hypertension, history of cardiovascular disease, BMI, eGFR, ACR, HbA1c, diabetes medications, interaction of HbA1c, and diabetes medications^9^, with a median AUC of 0.81. ADVANCE study enrolled 11,140 patients with type 2 diabetes aged ≥ 55 years and included eight predictors, such as ethnicity, eGFR, ACR, SBP, HbA1c level, diabetic retinopathy, blood pressure–lowering treatment at baseline, and waist circumference^20^, with the AUC value of 0.647 for new-onset albuminuria. Our model comprised persons with type 2 diabetes aged ≥ 40 years and used 11 predictors with an AUC value ranging 0.78–0.79 in the derivation set. In addition to traditional risk factors, our model considered variation in FPG and HbA1c. However, the definitions of outcomes among these studies had been determined by different biomarkers and cutoff points^9,11,20^, which may explain the performance of these CKD models. Our study’s definition was based on the guidelines of the National Kidney Foundation's Kidney Disease Outcomes Quality Initiative (NKF-KDOQI)^26^, which integrates eGFR and ACR.

As for the ESRD prediction model in persons with type 2 diabetes, the Building, Relating, Assessing, and Validating Outcomes (BRAVO) risk engine, using the data from the Action to Control Cardiovascular Risk in Diabetes (ACCORD) trial (n = 10,251), found four predictors, consisting of HbA1c, SBP, congestive heart failure (CHF) history, and blindness history with an AUC of 0.592 (0.459–0.619)^27^. In addition to the common factors of CKD and ESRD models, such as HbA1c and SBP, the predictors identified in BRAVO included late-stage complications such as CHF history and blindness history.

The risk equation of CKD Prognosis Consortium study was based on 28 countries and 34 multinational cohorts with 781,627 patients with type 2 diabetes with a mean age of 62 years. Their risk equations had a median AUC for 5-year predicted probability of 0.801 (interquartile range: 0.750–0.819) in a diabetes cohort for outcome of eGFR < 60 mL/min/1.73 m^2^. Using our study’s CKD definition, we used NRI and IDI to compare the equation of CKD Prognosis Consortium study with our scoring system, and the NRI of 0.189 indicates that our scoring system provided a slightly better reclassification of CKD (*p* < 0.001), whereas the IDI of 0.174 denotes that our scoring system achieved a slightly better discrimination improvement (*p* < 0.001).

### Clinical implication

The application of this prediction model is easy. Suppose that a health professional helps a 55-year-old (risk score = 3) men with diagnosed type 2 diabetes for 6 years (risk score = 2), had an eGFR of 80 mL/min/1.73m^2^ (risk score = 6), a ACR of 20 (risk score = 0), a triglyceride of 130 mg/dL (risk score = 0), a variation of HbA1c of 5% (risk score = 1), a variation of fasting blood glucose of 35% (risk score = 2), didn’t have a history of diabetes retinopathy at baseline (risk score = 0), is taking oral anti-diabetes medicine but no insulin use (risk score = 0) and is taking anti-hypertensive drug use (risk score = 1) to calculate his risk, and his sum risk score is 15 (Table 3). Thus the 1-, 3-, and 5-year risk of CKD will be 12.53%, 30.97%, and 43.62%, respectively (Supplementary Table S2).

### Strengths and limitations

The present study exhibited several strengths. First, this study introduced the first CKD prediction model in a relatively large group of patients with type 2 diabetes for Asians with a mean follow-up period of 3.8 years. Second, our study considered a wide range of potential risk factors, including 24 h dietary intake and medication use. The 24 h dietary variables included the percentages of total kilocalories from carbohydrate, protein, and fat intakes. The medication use included anti-diabetes, anti-hypertension, cardiovascular, and hyperlipidemia medications. Baseline visit-to-visit variation in HbA1c and FPG, renal function, and albuminuria play principal roles in our prediction model. The present study showed good discrimination capability for CKD risk score in Taiwanese persons with type 2 diabetes. Third, the definition of CKD outcome event adopted the 2012 NKF-KDOQI guidelines, with eGFR and ACR considered as factors, which is relatively rigorous. Finally, our study was based on the study subjects of a medical center. The value of the models derived from a single cohort is that it reduces the variation in measurements and patient care. Thus, the measurements of biomarkers were more standardized compared with those of multicenter studies.

The present study also encountered limitations. First, the outcome event we defined was based on one-day patient data below threshold, and we ignored the presence of the outcome event for > 3 months. However, we had adopted a definition of the CKD outcome by considering eGFR and ACR. The widely adopted definition of CKD is based on the 2013 Kidney Disease Improving Global Outcomes (KDIGO) guidelines^28^. It has been recognized the importance of integrating both GFR and albuminuria into CKD staging for providing more precise classification and more accurate prognostic information. Second, our study used the DCMP database, and the data came from a single center, which might have resulted in the condition in which the characteristics of our study sample were not representative of the subjects. Thus, our study findings may not be generalized to the entire population with diabetes. However, our results can be generalized to populations with diabetes and have similar characteristics as our study sample. Third, our study had the potential bias associated with losses to follow-up and competing risks. In the present study, all persons provided at least one assessment during follow-up period and the retention rate in the study endpoint was more than 90%, i.e., less than 10% (9.58%) of persons who died or lost to follow-up. In DCMP, care managers established good rapport with participants, thus the potential bias due to loss to follow-up may be minimized. As for the impact of competing risks, because DCMP dataset didn’t link with National Death Registry, we cannot use statistical analysis with competing risks. Due to much lower mortality rate compared with CKD incidence, the impact of competing risks may be small^29^. Fourth, the data used in the present were in 2002–2006 and few persons received GLP1R agonists or SGLT2i for contemporary use in 2021. Thus, the prediction model of the present study cannot be used to predict risk of incident CKD in patients receiving either GLP1R agonists or SGLT2i or both. Fifth, when applying this risk score in clinical practice, the management of missing data is an importance issue because clinicians might not have all of the covariates when submitting patient’s data. It may be needed to study how to solve this problem in future study. Last, the present study is a retrospective analysis of prospectively collected data and is subject to all the limitations of such a design, including no control over the nature and the quality of the measurements that were made, and incomplete, inaccurate data, or data measured in ways that are not ideal for the present study. For example, there was no biological variables such as relevant biomarkers of cystatin C, neutrophil gelatinase-associated lipocalin (NGAL, a new marker of kidney disease), etc., in addition to urine albumin-to-creatinine ratio (UACR). Instead, we considered relevant biomarkers that are routinely collected in clinical practice. Thus, our results can be easily applied in clinical practice.

## Conclusion

We set up a risk score system to predict CKD with variables routinely collected in daily clinical settings of a diabetes management system in persons with type 2 diabetes in Taiwan. Our score system consists of traditional risk factors, such as age, eGFR, and ACR, along with variables that were ignored in prior studies, including variations in HbA1c and FPG. The 1-, 3-, and 5-year CKD risk prediction models showed good prediction accuracy and discriminatory capability.

## Methods

### Data sources for derivation and validation sets

In 2001, the National Health Insurance Bureau in Taiwan established DCMP, and patients with confirmed diabetes mellitus disease based on the criteria of the American Diabetes Association were invited to enroll. DCMP aims to enhance the quality of diabetes care through providing continuous care and increasing the monitoring frequency to decrease diabetes-related complications. The participants are interviewed using a standardized questionnaire by a care manager upon enrollment. Information included prior or current disease status and lifestyle behaviors. All participants underwent tests such as blood, urine, and body measurements during follow-up visits every 3–4 months after their enrollment. This study included all identified patients who had been continuously enrolled in the DCMP program of a medical center in Taichung, Taiwan from 2002 until 2016.

The research design is a retrospective cohort study conducted on patients with a diagnosis of type 2 diabetes without CKD in 2002–2016 and who were enrolled in the DCMP database of a medical center in Taiwan. With an open cohort set-up, eligible patients with type 2 diabetes were allowed to enter and leave the cohort at any time point. The inclusion criteria were persons diagnosed as having type 2 diabetes based on the criteria of the American Diabetes Association without CKD and aged 20 years and older. The date of one year after entry to DCMP was defined as the index date, i.e., baseline. The study initially identified approximately 19,008 ethnic Taiwanese persons from the DCMP database. After the exclusion of 332 persons with type 1 diabetes and 2,719 study subjects with no index date or had an index date before the initial date of DCMP, 15,957 subjects were retained in the cohort. We further excluded study subjects aged younger than 40 years (n = 509) and persons with CKD at baseline (n = 3,555). To ensure the inclusion of patients with follow-up information or period, we excluded study subjects having one record on eGFR or ACR (n = 2,914) and those with a follow-up period of less than one year (n = 1,199), resulting in 7,780 eligible individuals. After further excluding patients with missing data for the duration of diabetes, smoking status, insulin injection, eGFR, ACR, HDL-C, retinopathy, TG, FPG variation, HbA1c variation, and hypertension drug use, 4,601 patients were retained in the data analysis. This study was approved by the Research Ethics Committee of China Medical University Hospital, and all methods were performed in accordance with the relevant guidelines and regulations. Informed consent was waived by the Research Ethics Committee of China Medical University Hospital (CMUH108-REC2-090), because the dataset used in this study consisted of de-identified secondary data released for research purposes.

### Data sources for external validation

The sample for external validation was from the national DCMP in Taiwan, comprising 5,795 ethnic Chinese patients with type 2 diabetes enrolling in DCMP during 2002–2004. The same inclusion and exclusion criteria as those for derivation and validation sets were applied. The follow-up assessment for outcome ascertainment was based on 2002–2011 datasets of National Health Insurance Research Database (NHIRD) implemented by Bureau of National Health Insurance (NHI), including inpatient and outpatient datasets, such as the patients’ birth date, gender, residential area, diagnosis, prescription of ambulatory claims. The coverage rate of NHI was approximately 99%. The CKD status was determined by ICD-9-CM codes (585) of NHIRD datasets.

### Measurements

The DCMP database comprises data for socio-demographic factors, lifestyle behaviors, and anthropometric measurements, such as age at baseline, gender, duration of diabetes, age of diabetes onset, smoking habits, alcohol drinking, exercise habit, body mass index (BMI), and nutritional intake. Smoking habits, alcohol drinking, and exercise habit were classified into two categories: yes or no. Nutritional intake included fat, protein, and carbohydrate and was acquired from patients’ 24 h recall through interview by a case management nurse. The food data from a single 24 h dietary intakes were linked with a food composition database to obtain nutrient information by probing the macronutrient content and deriving energy and macronutrient intakes from each food item. Then, the amount of consumption of each item was multiplied by its caloric content per serving to achieve the daily energy intake, and the caloric intakes for all food items were summed. Finally, we derived the percentage of total kilocalories from carbohydrate, protein, and total fat intakes. These variables were classified into three classes: insufficient (below adequate intake), sufficient (adequate intake), and over-intake (over tolerable upper intake levels). The ranges of carbohydrate between 50 and 60%, protein between 10 and 20%, and total fat between 20 and 30% were defined as normal. The number of days between the date of DCMP enrollment and diabetes onset was calculated to define the duration of diabetes. Then, the number of days was divided by 365 to convert the duration of diabetes as years. The age of diabetes was self-reported by patients.

### Laboratory examination

The biomarkers in the DCMP database include systolic blood pressure (SBP), diastolic blood pressure (DBP), HbA1c, FPG, low-density lipoprotein cholesterol (LDL-C), HDL-C, creatinine, total cholesterol, TG, GPT, eGFR, and ACR. Blood pressure was measured using an electronic device with the suitable size cuff (OMRON, HBP-9020, Japan). The patients’ blood was drawn from ante-cubital vein in the morning after an 8 or 12 h overnight fasting and sent for analysis within 4 h of blood collection. Whole blood of HbA1c was measured by boronate affinity high-performance liquid chromatography (Premier Hb9210™, Trinity Biotech Plc, IDA business Park, Ireland), plasma concentration of FPG was measured using glucose oxidase method (A&T Glucose Analyzer GA05, A&T, Tokohama, Japan), and serum concentrations of HDL-C, LDL-C, creatinine, TG, and total cholesterol were analyzed by a biochemical auto-analyzer (Beckman Coulter Synchron System, AU5800, Fullerton, CA, USA) at the Clinical Laboratory Department of CMUH. The intra-run CVs for HbA1c is below 2% and linearity is from 3.8% HbA1c to 18.5% HbA1c, ensuring accuracy for the whole population with diabetes. Inter- and intra-assay coefficients of variation (CVs) for FPG were both 4%. HDL-C and TG were measured in serum mode. TG levels were determined by an enzymatic colorimetric method. Inter- and intra-assay CVs for TG were 6.8% and 5%, respectively. HDL-C level was measured by a direct HDL-C method, and the inter- and intra-assay CVs were both 4.5%. LDL-C level was also measured by a direct LDL-C method, and inter- and intra-assay CVs were 4.5% and 3%, respectively. The albumin excretion rate was determined by UACR, urinary albumin (mg/dl) divided by creatinine (g/dl), measured from morning urine spot samples. Urinary creatinine (Jaffe’s kinetic method) and albumin (colorimetry bromocresol purple) were measured with an autoanalyzer (Beckman Coulter Synchron system, Lx-20, Fullerton, CA, USA). Inter-assay precision (coefficient of variation) was < 3.0% for both creatinine and albumin concentrations. UACR ranging from 30 mg/g to 300 mg/g was defined as microalbuminuria and above 300 mg/g was macroalbuminuria^28^.

eGFR was calculated by creatinine in the serum with the following formula: eGFR = 141 × minimum (serum creatinine/k, 1) ^α^ × maximum (serum creatinine/k, 1)^−1.209^ × 0.993^Age^ × (1.018 if female) × (1.159 if black), where k = 0.7, α = − 0.329 if female; k = 0.9, α = − 0.411 if male. BMI was derived from the formula weight (kg) ÷ (height)^2^ (m^2^). The calculation of CV, standard deviation over mean, used the first year measurements of SBP, DBP, HbA1c, and FPG for each patient after entry date. CV was used because this measure quantifies the relative variation by weighing the mean and further were adjusted for the number of patient measurements. The variation measures were derived when a person had two or more measurements in the first year after index date.

### Comorbidity at baseline

The present status of each specific comorbidity at baseline was ascertained from the DCMP database and self-reported by patients, and it was confirmed by the case manager from their electronic records of outpatient and inpatient care. Our study considered comorbidities, such as hypertension, hyperlipidemia, stroke, ischemic heart disease, angina, obesity, peripheral neuropathy, diabetes neuropathy, diabetes retinopathy, diabetic ketoacidosis, hypoglycemia, postural hypotension, hyperglycemic hyperosmolar nonketotic coma, vascular bypass, peripheral vascular disease, and amputation.

### Medications use at baseline

The use of medication for treatment at baseline was ascertained from the DCMP database and comprised anti-diabetes medication, anti-hypertension medications, cardiovascular medications, and hyperlipidemia medications. The anti-diabetes medication included two main categories: oral medication agents and insulin injection. Oral medication agents included seven categories: sulfonylurea, meglitinide, biguanide, α-glucosidase inhibitor, insulin sensitizer, dipeptidyl peptidase 4 inhibitor, and other compounds. Seven kinds of insulin injection were used: insulin aspart, regular insulin (RI), Neutral Protamine Hagedorn (NPH) insulin, levemir, lantus, and glucagon-like peptide-1 (GLP-1) analogue. All of the medications used were classified into two levels: yes or no.

### Outcome event

We identified patients who had incident CKD events from the DCMP database one year after their enrollment until 2016. The incident CKD cases were defined as having one of the following three conditions: ACR data greater than or equal to 300 (mg/g); both eGFR data less than 60 (ml/min/1.73 m^2^) and ACR data greater than or equal to 30 (mg/g); and eGFR data less than 45 (ml/min/1.73 m^2^)^30^.

### Statistical analysis

For continuous variables, we presented means and standard deviations (SDs), and for the categorical variables, we used proportions to describe the baseline characteristics of the study subjects in the deviation and validation sets. We developed a prediction model of CKD in the derivation set and evaluated the model’s predictive accuracy in the validation set by randomly assigning study subjects into derivation and validation sets at a 2:1 ratio. The development of the predictive model was based on the algorithm proposed by Framingham Heart study^30^.

Cox’s proportional hazards models with the hazard ratio (HR) with 95% confidence intervals were used to estimate the strength of associations between predictor variables and CKD, and the model-building steps used to select independent variables resulting in the “best” model included four steps. First, a careful univariate analysis of each variable was conducted. Second, if the variable in univariate analysis had a *p* value < 0.25, it was considered a candidate for our multivariable analysis^31,32^. Third, we set up a multivariable model with candidate variables and checked for collinearity. If the variables showed high collinearity, then we compared their significance and estimated their regression coefficients with those in univariate analysis. If a subset of variables indicated high correlations, i.e., multi-collinearity problem, only the variable that explained an outcome best was retained in the multivariate Cox model. In addition, only variables with *p* < 0.05 can be retained in the final model. Finally, after setting up a multivariate main-effect model, we checked the assumption of Cox’s proportional hazard model for all variables in our multivariate model and further assessed the interactions between independent variables.

We constructed the risk score function following the seven steps from the Framingham Heart study^30^**:** (1) parameters of the multivariable Cox’s proportional hazards model were estimated; (2) risk factors were grouped into categories, and the reference values (Wi) were decided; (3) a score was assigned for each category to determine the referent risk factor profile W_iREF_ in the scoring system; a base category for each risk factor was assigned a 0 score; (4) regression units were used to determine the distance from the base category to each category; (5) the constant B was set, and this number of regression units reflects 1 point in the final point system; (6) the number of points for each category of each risk factor was calculated, that is, Point_ij_ = (W_ij_ − W_iREF_)/B, where j is the category j; (7) prediction risks were determined for all possible total scores. The risk of CKD was calculated by the following equation: $$\hat P$$ = 1−*P*_0_(t)^exp^^(^$$^{{\sum }\beta_i \times X_i-\beta_i \times \bar{X}_i}$$^)^, where *P*_0_ is the baseline disease-free probability, β $$i$$ is the regression coefficient for X_i_, and X̅ is the mean level of X_i_.

To exploit the method of score assignment of Framingham Heart study, we classified all continuous variables considered in the analysis into categorical variables. The risk prediction model’s predictive accuracy was assessed by area under receiver operating characteristic (ROC) curve (AUC). The AUC presented the capability of the model to correctly discriminate the subjects into CKD or non-CKD cases. The range for AUC value was from 0 to 1. A value higher than 0.7 means that the model has a good discrimination. To assess the predictive capability of the risk prediction model, we categorized sum risk scores in the validation set into three subgroups and compared their cumulative incidence curves. The calibration of the model used goodness-of-fit to compare the observed and predicted events of CKD, which were tested by Hosmer–Lemeshow $\mathcal{X}^2$ method. For internal validation, we used 1000 times bootstrap resampling to correct the potential for overfitting or “optimism”.

We used multiple imputation method to impute missing data regarding the duration of diabetes, smoking, insulin injection, eGFR, ACR, HDL-C, retinopathy, TG, FPG variation, HbA1c variation, and hypertension drug in the sensitivity analysis. The same model’s discrimination and calibration in analysis can be assessed using the dataset without excluding the missing data. In addition, we perform the sensitivity analyses of stratification by sex and examining the interaction between sex and the other risk factors. We conducted all statistical analyses using SAS software, version 9.4 (SAS Institute Inc., Cary, NC, USA) (https://www.sas.com/en_us/legal/editorial-guidelines.html). Significance level was set at two-tailed *P* < 0.05.

## Supplementary Information

Supplementary Information.
