# Supplementary material for: Development and validation of a risk prediction model for chronic kidney disease among individuals with type 2 diabetes
Source: Sci Rep. 2022 Mar 21;12:4794. doi: 10.1038/s41598-022-08284-z (PMC8938464; doi:10.1038/s41598-022-08284-z)
Supplement: Supplementary file 1 — Supplementary Information. [file 41598_2022_8284_MOESM1_ESM.docx]

Supplementary table S1. Number of participants and CKD cases, incidence rates of CKD, and age- and gender-adjusted hazard ratios for baseline predictors in derivation set

| Variables | n | cases | Person-year | IR | HR (95% CI) | P-value for Cox’s assumption |
| --- | --- | --- | --- | --- | --- | --- |
| **Socio-demographic factors and behaviors** | | | | | | |
| Age (years) | 3067 | 786 | 11564 | 6797 | 1.05 (1.04, 1.06) | 0.33 |
| Duration of diabetes (years) | | |  |  |  |  |
| 0 | 752 | 141 | 2861 | 4928 | 1.00 |  |
| 1-5 | 1382 | 314 | 5408 | 5806 | 1.14 (0.94, 1.40) | 0.83 |
| >5 | 933 | 331 | 3295 | 10047 | 1.68 (1.37, 2.05) | 0.61 |
| **Renal function markers** | | | |  |  |  |
| eGFR (ml/min/1.73m^2^) |  |  |  |  |  |  |
| ≥90 | 1841 | 293 | 7463 | 3926 | 1.00 |  |
| 60-89 | 1226 | 493 | 4101 | 12022 | 2.36 (2.00, 2.78) | 0.13 |
| ACR (mg/g) |  |  |  |  |  |  |
| <30 | 2370 | 444 | 9409 | 4719 | 1.00 |  |
| ≥30 | 697 | 342 | 2154 | 15877 | 3.31 (2.87, 3.81) | 0.09 |
| **Lipid profiles** | | |  |  |  |  |
| HDL-C (mg/dL) | | |  |  |  |  |
| ≥40 (male)/ ≥50 (female) | 1351 | 295 | 4992 | 5910 | 1.00 |  |
| <40 (male)/ <50 (female) | 1716 | 491 | 6571 | 7472 | 1.33 (1.15, 1.53) | 0.22 |
| Triglyceride (mg/dL) |  |  |  |  |  |  |
| <150 | 1942 | 454 | 7509 | 6046 | 1.00 |  |
| ≥150 | 1125 | 332 | 4055 | 8188 | 1.54 (1.33, 1.78) | 0.20 |
| **Variation in glycemic factors** | |  |  |  |  |  |
| Variation of HbA1c (%) |  |  |  |  |  |  |
| ≤3.9 | 1021 | 211 | 4061 | 5196 | 1.00 |  |
| 4.0-8.0 | 1006 | 274 | 3732 | 7342 | 1.47 (1.23, 1.76) | 0.82 |
| >8.0 | 1040 | 301 | 3771 | 7982 | 1.73 (1.45, 2.07) | 0.15 |
| Variation of FPG (%) |  |  |  |  |  |  |
| ≤10.7 | 1014 | 187 | 3824 | 4890 | 1.00 |  |
| 10.8-20.3 | 1012 | 260 | 4053 | 6415 | 1.37 (1.13, 1.65) | 0.26 |
| >20.3 | 1041 | 339 | 3687 | 9194 | 2.02 (1.69, 2.42) | 0.15 |
| **Baseline diabetes-related disorders or complications** | | | |  |  |  |
| Diabetes retinopathy |  |  |  |  |  |  |
| No | 2467 | 587 | 9550 | 6147 | 1.00 |  |
| Yes | 600 | 199 | 2014 | 9881 | 1.54 (1.31, 1.81) | 0.14 |
| **Medication use** |  |  |  |  |  |  |
| Insulin use |  |  |  |  |  |  |
| No | 2822 | 684 | 10726 | 6377 | 1.00 |  |
| Yes | 245 | 102 | 837 | 12180 | 2.17 (1.76, 2.67) | 0.70 |
| Hypertension drug use |  |  |  |  |  |  |
| No | 1942 | 422 | 7467 | 5652 | 1.00 |  |
| Yes | 1125 | 364 | 4097 | 8884 | 1.39 (1.21, 1.61) | 0.44 |

CKD: chronic kidney disease; eGFR: estimate glomerular filtration rate; ACR: albumin to creatinine ratio; HDL-C: High-density lipoprotein; variation of FPG: variation of fasting plasma glucose.

IR: incidence density rate (number of incident cases/person-years*100,000); HR: hazard ratio; CI: confidence intervals.

**Supplementary table S2.** 1-,3-,5-year estimated risk for CKD of each possible sum points

| Point total | Estimate of 1-year risk | Estimate of 3-year risk | Estimate of 5-year risk |
| --- | --- | --- | --- |
| 0 | 1.08% | 2.96% | 4.54% |
| 1 | 1.27% | 3.49% | 5.34% |
| 2 | 1.50% | 4.11% | 6.28% |
| 3 | 1.78% | 4.84% | 7.39% |
| 4 | 2.10% | 5.70% | 8.67% |
| 5 | 2.48% | 6.70% | 10.17% |
| 6 | 2.92% | 7.88% | 11.91% |
| 7 | 3.44% | 9.25% | 13.93% |
| 8 | 4.06% | 10.84% | 16.25% |
| 9 | 4.78% | 12.68% | 18.92% |
| 10 | 5.63% | 14.82% | 21.96% |
| 11 | 6.62% | 17.27% | 25.41% |
| 12 | 7.78% | 20.09% | 29.29% |
| 13 | 9.13% | 23.29% | 33.62% |
| 14 | 10.70% | 26.91% | 38.41% |
| 15 | 12.53% | 30.97% | 43.62% |
| 16 | 14.64% | 35.49% | 49.21% |
| 17 | 17.07% | 40.44% | 55.12% |
| 18 | 19.85% | 45.81% | 61.22% |
| 19 | 23.02% | 51.54% | 67.37% |
| 20 | 26.61% | 57.54% | 73.40% |
| 21 | 30.64% | 63.68% | 79.11% |
| 22 | 35.12% | 69.81% | 84.30% |
| 23 | 40.04% | 75.74% | 88.80% |
| 24 | 45.38% | 81.26% | 92.49% |
| 25 | 51.09% | 86.19% | 95.31% |
| 26 | 57.07% | 90.38% | 97.32% |
| 27 | 63.20% | 93.72% | 98.61% |
| 28 | 69.34% | 96.21% | 99.37% |
| 29 | 75.28% | 97.91% | 99.75% |
| 30 | 80.85% | 98.97% | 99.92% |
| 31 | 85.83% | 99.55% | 99.98% |
| 32 | 90.08% | 99.83% | 99.99% |
| 33 | 93.49% | 99.95% | 100.00% |
| 34 | 96.05% | 99.99% | 100.00% |
| 35 | 97.81% | 100.00% | 100.00% |
| 36 | 98.91% | 100.00% | 100.00% |
| 37 | 99.52% | 100.00% | 100.00% |

|  | Non-event | | | | | |
| --- | --- | --- | --- | --- | --- | --- |
|  |  | Risk in our model | | | |  |
|  |  | <16% | 16-44% | ≥44% | Total |  |
| Risk in CKD Prognosis Consortium model | <16% | 1204 33.62% | 1552  43.34% | 604  16.87% | 3360  93.83% |  |
|  | 16-44% | 0  0.00% | 70  1.95% | 132  3.69% | 202  5.64% |  |
|  | ≥44% | 0  0.00% | 2  0.06% | 17  0.47% | 19  0.53% |  |
|  | Total | 1204  33.62% | 1624  45.35% | 753  21.03% | 3581  100.00 |  |

|  | Event | | | | | |
| --- | --- | --- | --- | --- | --- | --- |
|  |  | Risk in our model | | | | |
|  |  | <16% | 16-44% | ≥44% | Total |  |
| Risk in CKD Prognosis Consortium model | <16% | 77  7.55% | 278  27.25% | 306  30.00% | 661  64.80% |  |
|  | 16-44% | 0  0.00% | 47  4.61% | 265  25.98% | 312  30.59% |  |
|  | ≥44% | 0  0.00 | 5  0.49% | 42  4.12% | 47  4.61% |  |
|  | Total | 77  7.55% | 330  32.35% | 613  60.10% | 1020  100.00 |  |

Supplementary table S3. The sensitivity analysis for net reclassification improvement

The performance of the equation published by Nelson et al. using multinational cohorts from the CKD Prognosis Consortium (CKDPC) was compared with our risk scoring system (Nelson RG, 2019). The net reclassification improvement (NRI) method quantified the amount of correct reclassification of CKD events between our new model and CKDPC equations. The integrated discrimination improvement (IDI) method quantified the extent that our new model increases the likelihood of CKD events and decreases the likelihood of non-CKD events (Pencina MJ, 2010). Positive values of NRI and IDI indicated that compared with CKDPC equations, the new model has better classification capability, and a large value means excellent discriminability capability. CKDPC equation: P=1 – exp {-5^1.212595 × exp [-4.56736 + 0.1086255 × (age/5 – 11) + 0.1130331 × (if female) + 0.1475446 × (if black) + 0.268425 × (15 – min(eGFR, 90)/5) – 0.1160029 × max(0, eGFR-90)/5 + 0.3073098 × (if has history of CVD) + 0.0841638 × (HbA1c –7) + 0.0909167 × (if insulin use) – 0.1855329 × (if no DM medication use) + 0.035993 × (HbA1c –7) × (if insulin use) + 0.0610776 × (HbA1c –7) × (if no DM medication use) + 0.0310965 × (if ever smoking) + 0.4748296 × (if hypertensive) + 0.0645142 × (BMI/5-5.4) + 0.5715969 × (log10ACR – 1)]};

Nelson RG, Grams ME, Ballew SH, et al. Development of Risk Prediction Equations for Incident Chronic Kidney Disease. *JAMA.* 2019;322(21): 2104-2114.

Pencina MJ, D'Agostino RB, Vasan RS. Statistical methods for assessment of added usefulness of new biomarkers. *Clin Chem Lab Med.* 2010;48(12): 1703-1711.

Supplementary table S4. Parameter estimates of regression coefficient and hazard ratios of predictors for CKD from the final multivariate Cox’s proportional hazards model stratified by sex

|  | Female | |  | Male | |
| --- | --- | --- | --- | --- | --- |
| Risk factor | $\hat{\boldsymbol{\beta}}$($\hat{\boldsymbol{SE}}$) | HR (95% CI) |  | $\hat{\boldsymbol{\beta}}$($\hat{\boldsymbol{SE}}$) | HR (95% CI) |
| **Socio-demographic factors** |  |  |  |  |  |
| Age | 0.03 (0.01) | 1.03 (1.02, 1.04)*** |  | 0.03 (0.01) | 1.03 (1.02, 1.04)*** |
| Duration of type 2 diabetes (years) (<1 year as reference) | | |  |  |  |
| 1-5 | -0.01 (0.12) | 0.99 (0.78, 1.25) |  | 0.12 (0.12) | 1.13 (0.90, 1.43) |
| >5 | 0.17 (0.12) | 1.19 (0.93, 1.51) |  | 0.19 (0.12) | 1.21 (0.94, 1.54) |
| **Renal function markers** |  |  |  |  |  |
| eGFR (≥90 mL/min/1.73m^2^ as reference) | 0.90 (0.10) | 2.46 (2.02, 3.00)*** |  | 0.77 (0.10) | 2.15 (1.78, 2.60)*** |
| ACR (<30 mg/g as reference) | 0.86 (0.09) | 2.36 (1.99, 2.80)*** |  | 1.18 (0.08) | 3.25 (2.75, 3.83)*** |
| **Lipid profiles** |  |  |  |  |  |
| HDL-C (male≥40 / female≥50 mg/dl as reference) | 0.13 (0.09) | 1.14 (0.95, 1.37) |  | 0.15 (0.08) | 1.16 (0.99, 1.37) |
| Triglyceride (<150 mg/dL as reference) | 0.22 (0.09) | 1.24 (1.04, 1.48)* |  | 0.28 (0.09) | 1.33 (1.11, 1.58)** |
| **Variation in glycemic factors** |  |  |  |  |  |
| Variation of HbA1c (%), (≤3.9% as reference) | | |  |  |  |
| 4.0-8.0 | 0.11 (0.11) | 1.11 (0.90, 1.38) |  | 0.21 (0.11) | 1.24 (1.00, 1.54) |
| >8.0 | 0.18 (0.12) | 1.19 (0.94, 1.52) |  | 0.38 (0.12) | 1.46 (1.16, 1.84)** |
| Variation of fasting plasma glucose (%), (≤10.7% as reference) | | |  |  |  |
| 10.8-20.3 | 0.14 (0.12) | 1.15 (0.91, 1.44) |  | 0.10 (0.12) | 1.11 (0.88, 1.40) |
| >20.3 | 0.45 (0.13) | 1.57 (1.22, 2.01)*** |  | 0.30 (0.12) | 1.36 (1.06, 1.73)* |
| **Baseline diabetes-related disorders or complications** | | |  |  |  |
| Diabetes retinopathy | 0.21 (0.10) | 1.24 (1.01, 1.50)* |  | 0.41 (0.10) | 1.51 (1.25, 1.82)*** |
| **Medication use** |  |  |  |  |  |
| Insulin use | 0.31 (0.14) | 1.37 (1.05, 1.79)* |  | 0.41 (0.14) | 1.51 (1.15, 1.98)** |
| Hypertension drug use | 0.20 (0.09) | 1.22 (1.03, 1.45)* |  | 0.21 (0.08) | 1.24 (1.05, 1.46)* |

CKD: chronic kidney disease; eGFR: estimate glomerular filtration rate; ACR: albumin to creatinine ratio; HDL-C: High-density lipoprotein; variation of FPG: variation of fasting plasma glucose; HR: hazard ratio; CI: confidence intervals.

*:p<0.05; **:p<0.01; ***:p<0.001.

**Supplementary Figure 1.** Flowchart of recruitment procedures for the CKD model.

19,008 individuals enrolled in the DCMP of CMUH

4,601 participants were included for analysis.

15,957 individuals with type 2 diabetes in the DCMP of CMUH.

Excluding n=3,051
Patient without index date or index date before 2001/11/01 (n=2,719)
Patients with type 1 diabetes (n=332)

Excluding n=8,177

Age < 40 patients with type 2 diabetes (n=509)

Patients with CKD at baseline (n=3,555)

Measurements of eGFR or ACR less than 2 (n=2,914)

CKD incidence within 1 year after entry (n=1,199)

Derivation Set

n=3,067

Validation Set

n=1,534

7,780 subjects were eligible

Excluding n=3,179

Without duration of diabetes, smoke, insulin injection, eGFR, ACR, HDL-Cl, retinopathy, triglyceride, FPG variation, HbA1c variation, hypertension drug use

Random allocation of 2:1 ratio

(A) (B) (C)


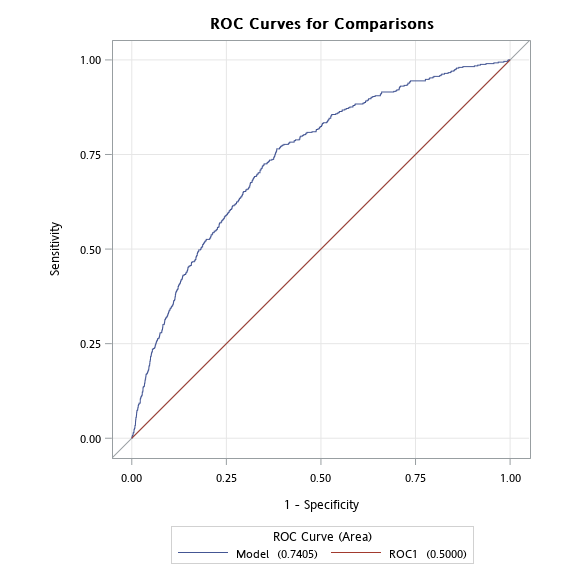

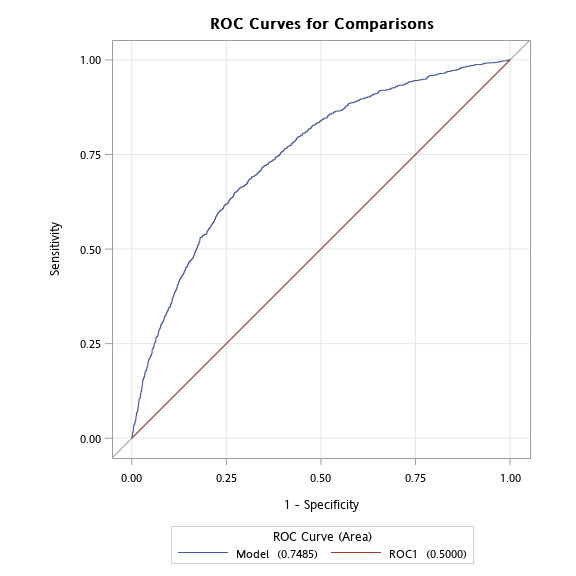

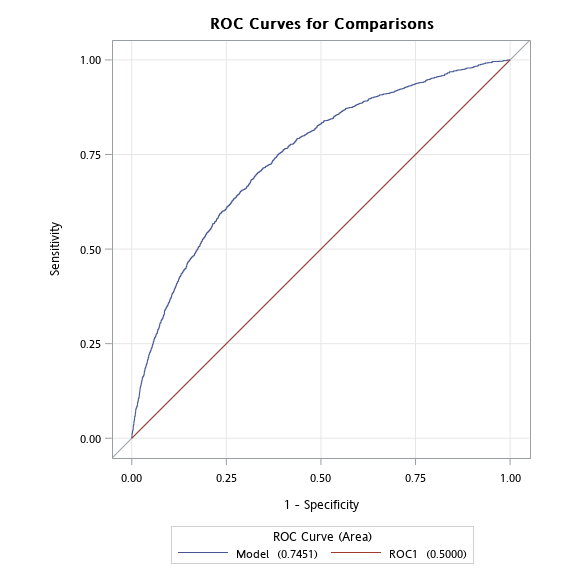


**Supplementary Figure 2**. Receiver operating characteristic curves (ROCs) for 1-year (A), 3-year (B), and 5-year (C) CKD risks in sensitivity analysis.

(A) (B) (C)

**Supplementary Figure 3.** Predicted versus oberserved CKD cases according to deciles of 1-year (A), 3-year (B), and 5-year (C) CKD risks in sensitivity analysis.
